# Cell-Based Biosensors: Electrical Sensing in Microfluidic Devices

**DOI:** 10.3390/diagnostics2040083

**Published:** 2012-12-06

**Authors:** Katrine Kiilerich-Pedersen, Noemi Rozlosnik

**Affiliations:** Department of Micro- and Nanotechnology, Technical University of Denmark, Oersteds Plads 345 East, DK-2800 Kongens Lyngby, Denmark; E-Mail: katk@nanotech.dtu.dk

**Keywords:** biosensor, microfluidics, mammalian cells, electrochemical impedance spectroscopy, medical diagnostics

## Abstract

Cell-based biosensors provide new horizons for medical diagnostics by adopting complex recognition elements such as mammalian cells in microfluidic devices that are simple, cost efficient and disposable. This combination renders possible a new range of applications in the fields of diagnostics and personalized medicine. The review looks at the most recent developments in cell-based biosensing microfluidic systems with electrical and electrochemical transduction, and relevance to medical diagnostics.

## 1. Introduction

Emerging pathogenic viruses or resistant bacteria in animals more frequently make the headlines than a decade ago. In a global community the outbreak of disease is a source of worry for the population, and the increased interest has indeed emphasized the need for new effective diagnostic screening solutions for point of care (POC) testing.

POC is an emerging field within medical diagnostics and disease monitoring, and eventually disease control. Integration of nanomaterials, microfluidics, automatic samplers, and transduction devices on a single chip provides many advantages for POC devices such as biosensors. Making use of specially designed microsystems [1], patients can be monitored continuously at bed side or at the general practitioner, and save precious time in commuting between home, doctor and hospital. The technological advancements in cell-based biosensors have accelerated the R&D in POC devices.

Conventional detection methods in medical diagnostics, such as polymerase chain reactions (PCR) and enzyme linked immunosorbent assays (ELISA), or whole animal testing are time consuming, labour intensive and expensive [2,3]. Accordingly, it is important to develop effective screening tools to detect, control and confine the spread of biohazards.

Cell-based biosensors—with living cells as the recognition element—are characterized by high sensitivity, selectivity and rapid response. They are very versatile and thus applicable in different fields such as food safety, environmental monitoring, drug screening and medical diagnostics. Cell-based biosensors are able to measure functional information and in this way help us to understand the cellular mechanisms behind particular diseases to improve the development of targeted treatment.

Cell-based biosensors can be constructed to detect the response of a single cell, a cell layer, or a network of cells. The mammalian cells are the most common recognition elements in the microsystems, allowing physiologically relevant studies of a cellular response to one or more compounds, or effects.

Several transducer methods are used for the recognition events including optical absorption and fluorescence, acoustic detection, surface plasmon resonance, electrical and electrochemical methods. Electrical signal detection can readily be integrated in biosensors and thus is an attractive alternative to other detection methods.

This review will focus on the recent developments of cell-based biosensing in microfluidic systems with electrical and electrochemical transduction that are relevant to medical diagnostics without aspiring to completeness. Readers interested in more general aspects of cell-based biosensors are directed to several other excellent review articles [4,5,6,7,8].

## 2. Device Considerations

For the new generation of POC diagnostics, sensors have to be producible at reasonable cost and fulfill high standards to outmatch the conventional techniques in medical diagnostics. Cell-based sensors are—by nature—very specific and sensitive giving a rapid response. Careful design considerations regarding the microfluidic channel network, electrode system, and selection of materials can further improve the microdevice significantly while maintaining them at low cost.

### 2.1. Microfluidic Systems

The microscale channels in a microfluidic device enable the use of extremely small volumes of expensive chemicals and low concentration of analyte. Since most of the bio- and chemical reactions are determined by diffusion of the molecules to the adequate places, the short distances in a microsystem permit the rapid detection by reducing the diffusion times. Both the mass and heat transport are faster in a microsystem, allowing a quasi-equilibrium state for the biochemical processes. A variety of microstructures can be used for optimization of transport processes, e.g., vortices, pillars, or herringbone.

A defined microfluidic environment is convenient for cellular studies because the physiological and electrical responses of single cells can be detected. All these desired properties of microfluidic devices are favourable in many biological and medical applications [9].

The majority of microfluidic cell culture systems are designed for adherent cells [10,11], as these are the dominant cell types used in biological studies. Besides, the handling and perfusion of fresh media for culturing of adherent cell types is much easier. Recently, a cell culturing microfluidic reactor for culturing both adherent and non-adherent cells was presented. It allowed precise control of pH, oxygen, nutrition and temperature, and sustained the biochemical microenvironment of the cells, while supplying nutrients to the cells by diffusion controlled processes [12]. By producing miniature electrodes on the surface of the microchannels, electrical or electrochemical signals from the cells could be recorded in real time.

### 2.2. Materials for Fabrication

The choice of materials for fabrication of microfluidic devices is a very important factor. Since the 1990s, several studies have originated on interfacing materials with living cells, with the goal of detecting biocomponents or biological processes. The considerations include biocompatibility of the materials, transparency for microscopy, and wettability for aqueous liquid handling [13,14]. Initially, silicon and glass were the most attractive materials for microdevices due to well developed fabrication technologies for semiconductors and micro-electro-mechanical-systems (MEMS).

More recently, polymers have gained popularity, because both the polymer material and the polymer manufacturing techniques (injection molding and hot embossing, replica molding, casting) are inexpensive [15]. Several polymers have physical, mechanical, and chemical properties that meet the demands for biosensing, for example polydimethylsiloxane (PDMS), polymethylmethacrylate (PMMA), polyethylene diacrylate (PEG-DA), polycarbonate, cyclic olefin copolymers (COC).

PDMS is typically employed for prototyping because of its many excellent properties (including gas permeability, transparency, flexibility, and biocompatibility). The surface of native PDMS is hydrophobic, but can be made hydrophilic by surface modification with plasma, UV/ozone, or corona discharge. The oxidized surface remains hydrophilic if it stays in contact with water, and can be modified further by treatment with functionalized silanes [16]. PDMS seals reversibly to a variety of materials such as glass, hard plastic, silicon, flat metal, photoresist, and native PDMS.

Mass production of polymeric devices is commonly done by the injection moulding technique using COC [17,18], and only few polymers are suitable for production of microscale structures with injection molding. COC is a thermoplastic polymer with desirable physical and chemical properties, such as high optical transmittance, low water absorption, biocompatibility and high chemical stability in aqueous media, alkaline, acidic, and polar solvents. Hence, this material is particularly attractive for disposable, cost effective, reliable microfluidic devices with lab-on-a-chip applications.

The novel material Graphene has emerged as an attractive candidate for the biointerface [19,20]. Graphene is a two dimensional sheet of hybridized carbon atoms arranged in a honeycomb lattice, and it exhibits several superior and atypical properties owing to a unique combination of its crystallographic and electronic structure. Due to these properties, it can be a sensitive platform for interfacing with biological cells to detect intra- and extracellular phenomena, including cellular excretion and potential modulation of the cell membrane. Although these applications are lacking of maturity, cell interfaced graphene devices can open avenues for diagnostics and single cell analysis in the future [20].

### 2.3. Sensing Unit

A major cost factor in electrical and electrochemical sensors is the sensing unit. Electrodes are typically made in a noble metal, involving extensive production steps and cleanroom facilities. Afterwards, surface treatments with a bio- or biocompatible material are required to attain biocompatibility with living cells. For cell-based applications the surface of the metal electrodes can be covered with an extracellular matrix protein to improve cell adherence.

Conductive polymer electrodes present an alternative to noble metals. In addition to low material cost, electrode fabrication is inexpensive, and the electrodes are easily functionalized [21]. The use of conductive polymers as supporting materials in microfluidic systems is well established. However, the electronic sensing units in most microdevices are fabricated from metallic conductors such as platinum or gold.

Biocompatibility and functionalization of electrodes is of great importance in cell-based biosensor applications, where cells are immobilized directly on the electrode surface. The conductive polymer poly(3,4-ethylenedioxythiophene) (PEDOT) has high environmental stability, biocompatibility, transparency to visible light and ease of processing [21]. This polymer has shown high potential and superior quality [22,23], and it has been employed in several biosensor microdevices [24,25].

**Figure 1:**
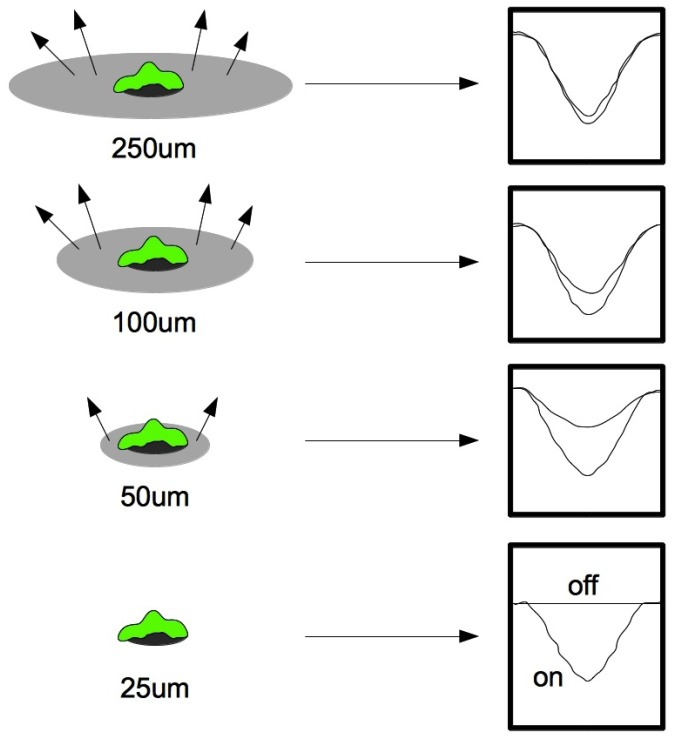
Electrochemical current response of microelectrodes for the presence of a single cell. The larger electrodes were incapable of resolving a single cell, whereas the use of small electrodes produced a clear change in the electrochemical impedance spectroscopy signal.

In general, the electrode design has major impact on the electrical readout, and the sensing unit should be designed and optimized in accordance with the application. The demands for studies of the dynamics of a cell culture on a conductive polymer interdigitated electrode array [25] are different than the demands for single cell investigations on an Au microelectrode array [26], or electrical characterization of single cells on polysilicon wires [27,28]. Figure 1 illustrates the electrochemical current response of microelectrodes for the presence of a single cell, hence emphasizing the effect of electrode design on the electrical signal.

### 2.4. Microfluidic Design

Microfluidic technology is creating powerful tools for cell biologists to control the complete cellular microenvironment, leading to new questions and new discoveries [29].

For cellular analysis it is often necessary to culture the cells. The standard methods (e.g., culture flasks) do not represent a natural environment. Microfluidic culturing systems for adherent and non-adherent cells allow for precise control of the environment and diffusion based feeding, where important chemicals are not flushed away as it can be the case in conventional cell culture flasks [12].

At micrometer scale, we have a detailed understanding of fluid behaviour, which confers unique potential to microfluidic gradient generating methods. Laminar flow based micro gradient generation devices are used for diffusive mixing of two or more parallel laminar streams of different composition to generate molecular gradients. Gradients generated in these types of devices will maintain their shape in time and space at constant flow rate. The analysis of cell migration in different regions of both chemotactic and haptotactic gradients [30] is relevant for the improved understanding of among others wound healing, cell invasion, and tumour progression.

An intelligent microfluidic cell culture system allows precise control of cell adhesion by temperature changes [31]. The shear stress dependent cell detachment process was investigated in five individual microchannels, where surfaces were coated with the well known temperature responsive polymer Poly(N-isopropylacrylamide) (PIPAAm). To estimate the interaction between cells and the PIPAAm layer, the cells were exposed to a flow in the microchannels, and the shear stress generated by this flow was used as a key factor for cell detachment. The proposed device could be used to assess the possible interaction between the cells and the PIPAAm layer with a potential application in tissue engineering.

## 3. Principles of Transduction

The feasibility of sensors that can convert the cellular signals from living cells to electrical signals in real time depends especially on cost, usability, sensitivity, and specificity. Real time sensing provides live information regarding the state of the cells, allowing for precise control. This is of great importance in POC applications where rapid response is crucial, or in long term cytotoxicity studies. When cell viability has a relevance (rather than the cell count) real time sensing is advantageous. The three principles of transduction described here enable real time sensing.

### 3.1. Electrochemical Methods

Electrochemical sensors produce an electrical signal in response to an electrochemical reaction between an analyte and the surface. Direct electrochemical signal detection is preferable, because the use of a simple set of electrodes greatly reduces the complexity, size and costs—factors that are typically associated with other methods, such as optical detection.

Buchinger *et al.* have evaluated the chrono-amperometric detection method in a reporter gene assay based on the bacterial response in comparison with standardized methods [32]. It was shown that the chrono-amperometric detection—under optimized electrochemical conditions—is sufficiently sensitive with a limit of detection that is comparable to the respective ISO-standard.

Among cell-based assays, impedimetric sensors have attracted attention for the high sensitivity, reliability and simplicity [33,34,35,36,37,38]. Electrochemical impedance spectroscopy combined with microelectrode arrays provide a platform for label free detection of the cellular response to different drugs or pathogens [39,40]. The interaction between a cell monolayer and the electrode surface can be monitored in real time by applying a small amplitude alternate-current (AC) electric field. Cells are essentially non-conducting at low frequencies and the cell membrane offers a significant barrier to current flow, so that the impedance of the system is an indication of the cell volume or size [41]. Average alterations in the three dimensional shape of cells is then computed by integrating over a monolayer of hundreds or thousands of cells. The measured impedance reflects changes in the attachment and morphology of cells, and reaches its maximum when the electrode is completely covered [25,42,43].

### 3.2. Field Effect Devices

**Figure 2:**
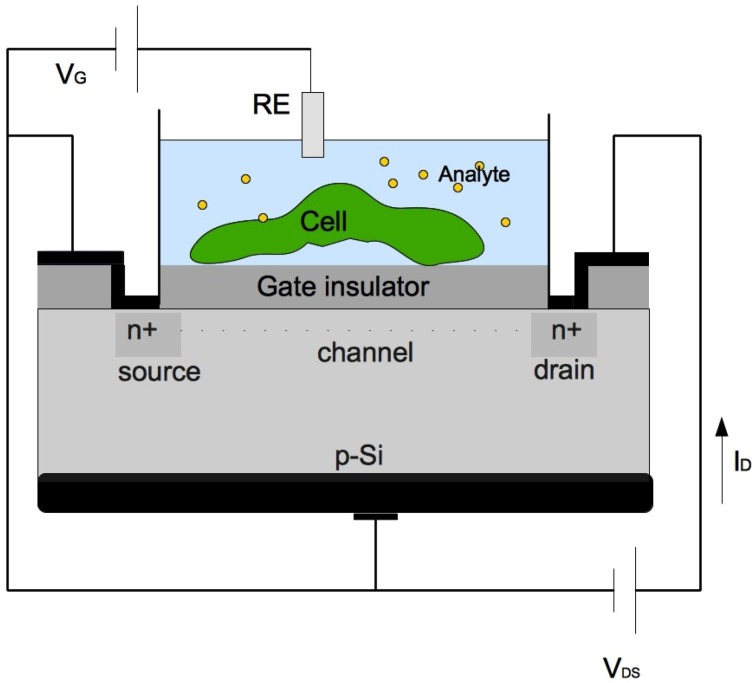
Cell/transistor hybrid. The open gate area of the FET is completely covered by one cell as indicated in the schematics (RE: reference electrode; VG: gate voltage; VDS: drain-source voltage; ID: drain current).

Field effect devices use an electric field to control the conductivity of a semiconducting material. The integration of living cells together with silicon field effect devices challenges a new generation of biosensors and bioelectronic devices. Among the variety of proposed concepts, the integration of living cells together with a silicon chip consisting of an array of (bio)chemical and/or electrophysiological transducers, based on a field-effect electrolyte/insulator/semiconductor system is one of the most attractive approaches. The cell/transistor hybrid is obtained by direct coupling of a single cell or cell system to the gate insulator of a field effect transistor (FET) (Figure 2). This system is very sensitive to any kind of electrical interaction at or nearby the gate insulator/electrolyte interface.

The state of a single cell or cell system can be monitored by means of various methods, such as (i) utilisation of the metabolism of cells like growth, toxicity, extracellular pH, redox potential, concentration of ions, *etc.*, (ii) utilisation of specific features of electrogenic cells, e.g., neuronal cells, or muscle cells by measuring the extracellular potentials [44,45].

### 3.3. Light Addressable Potentiometric Sensors

**Figure 3:**
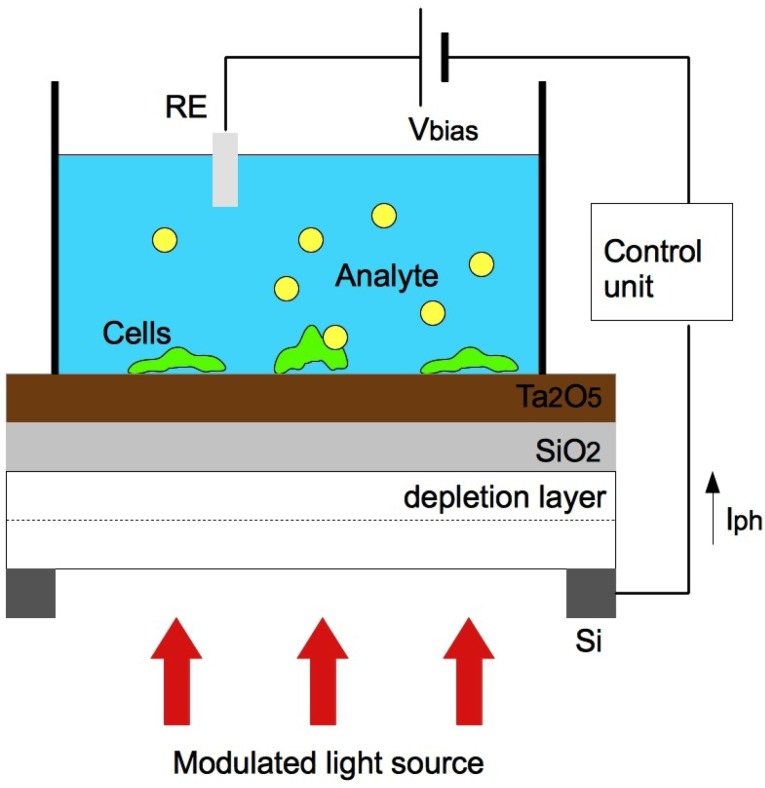
Schematic set up of a LAPS device with living cells and light sources (RE: reference electrode; Vbias: bias voltage; Iph: generated photocurrent).

Light addressable potentiometric sensors (LAPS) use light to activate carriers in a semiconducting material. LAPS (Figure 3) are popular in chemical and biological applications, primarily because of high spatial resolution and because the measuring point can be selected by a scanning light beam [46].

Electrochemical interactions between the transducer surface and the analyte generate surface potentials. They are then added to the applied DC bias voltage. To detect the gate insulator surface potential, the LAPS is illuminated with a modulated light source using laser beam or light emitting diodes (LED) in portable devices. The light source induces an AC photo current, which is measured as the sensor signal.

The light pointer in a LAPS device can be addressed at individual cells in culture, allowing for single cell analysis on chip. Although many cells are cultured on a chip surface, only the cell(s) in the illuminated area is interrogated.

## 4. Applications

Cell-based sensors are applicable in many areas of medical diagnostics, and have a large potential in the emphasized fields.

### 4.1. Pathogens and Toxins

**Figure 4:**
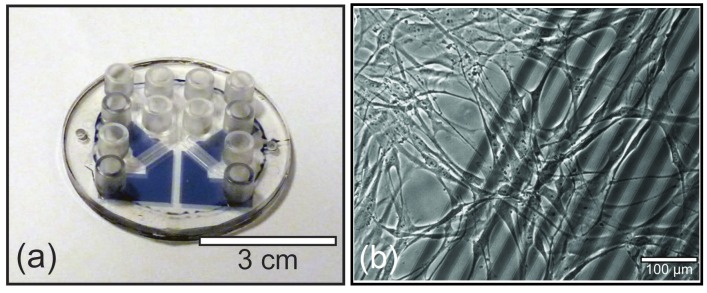
Cell-based biosensor. (**a**) All polymer microdevice with two functional channels fabricated in TOPAS (uncolored) and PEDOT:TsO (blue). (**b**) Healthy cells cultured on PEDOT:TsO microelectrodes in the biosensor.

Electrochemical impedance spectroscopy measured on cells cultured on a microelectrode array is very sensitive to small changes in the cell membrane capacitance and resistance (Figure 4). These parameters are good indicators for the well being of cells at a given cell morphology. The real time detection of cell impedance gives an efficient and rapid technique for non-invasive monitoring of the response of human cells in culture to the challenge of a virus infection [25,47,48], or a drug [49,50].

Cardiac cell-based biosensors have been used to study toxicity induced by a drug or heavy metal ions [51,52,53]. The toxic effects of ions lead to clear alterations in the action potential and were detectable within fifteen minutes [52].

### 4.2. Single Cell Analysis

Single cell analysis is of importance in certain aspects of medicine. Regeneration of neural tissue is very complex and requires multiple signals for axonal regrowth and functional recovery of damaged nerve tissue [54]. Microsystems allow cultures to be seeded at very high density in two or three dimensions to achieve cell-cell contact and generate an environment more anatomically similar to living tissue. Scaffolds of electrically conducting fibers immobilized with neural growth factor on PPy present multiple stimuli simultaneously and are attractive for neural cells, serving as a guidance and supporting neurite formation and outgrowth [55]. Understanding the complex signaling of neurites is a key in the process of understanding neurite migration and differentiation. A quantitative measure of cellular transmitter release by neuronal cells was measured by electrochemical techniques. The cells were trapped in a closed microchip close to a band of microelectrodes [56].

### 4.3. Drug Discovery

Cell-based biosensors with intact living cells as the sensor allow for screening of virtually any drug. Synchronization of the cell cycle by serum starvation is a common technique to enhance the cellular response [57].

Wound healing is complex process and it can be examined and accelerated in a biosensor [58].

**Figure 5:**
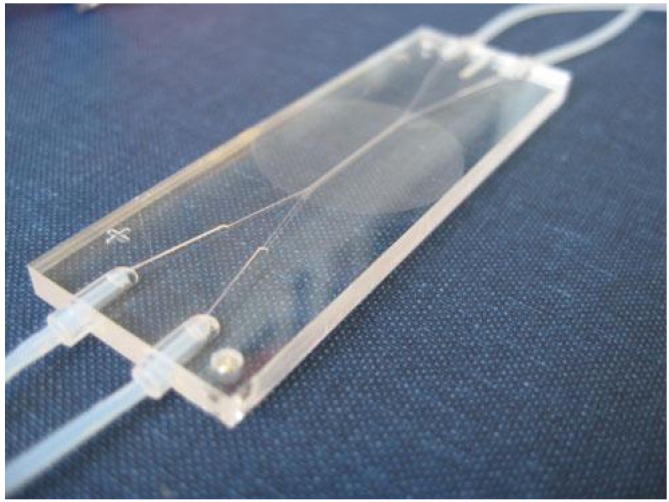
Microfluidic device for studies of chemotactic cell migration. The chip was fabricated in PMMA and contained separate channels for cells and chemoattractants. This type of assay has application in drug screening [64].

Migration assays and invasion assays are well suited for drug screening by rapidly and quantitatively measuring cell movement and the ability to traverse physical barriers. The microfluidic technology presents an appealing strategy to control fluid flow necessary to generate gradients on a scale suitable for cellular migration studies. New gradient generating methods to study chemotactic [59,60,61] or haptotactic [62] cell migration are considered more advantageous than conventional methods such as the transwell assay [63]. A novel microfluidic migration device imitates the physiological conditions of cell transmigration (Figure 5) [64]. The principles of a shear flow chamber were combined with the transwell assay in a microfluidic system that closely resembles the natural environment of migrating immune cells. Microchannels were physically separated by a porous membrane, and the cells actively migrated towards a pore in response to a chemotactic gradient, similarly to extravasation from blood vessels to underlying tissue. In this work, the transmigrating cells were mapped using fluorescent imaging, but an electrode array could be implemented to count migrating immune cells by electrochemical impedance spectroscopy.

### 4.4. Cancer

Early-stage diagnosis of cancer is the critical factor for treatment and patient survival. Primary tumors can often be removed with advanced therapies and drugs. Unfortunately, many cancers are diagnosed after cancer cells have escaped from the primary tumor site, circulated with the blood stream to form secondary tumor sites throughout the body. Currently, invasion and metastases of tumors are the main reasons for patient mortality. The invasion properties of different cell lines were studied in a cell-based biosensor by electrical impedance spectroscopy [65]. New techniques are required for diagnosis and monitoring. Sensors with ultra-low detection limits can be targeted towards emerging cancer biomarkers (indicators of tumor growth), cancer cells in circulating blood (indicators of metastasis) and to determine drug effectiveness at various target sites. Precise counting of breast cancer cells was achieved on a gold microelectrode array via label free electrochemical impedance spectroscopy based detection at a single cell level [66,67].

## 5. Conclusion and Outlook

Elegant multidisciplinary research collaborations have provided new horizons for cell-based sensing in medical diagnostics. Lately, we have seen a wide variety of microsystem based sensors with many different clinical applications such as high throughput drug screening. Cell-based biosensors further have application in tissue engineering for regeneration of highly organized tissues facilitated by the patterning technologies. Some of the new cell-based biosensing systems will meet the requirements of high specificity and sensitivity, low cost, simplicity, and rapid readout—they raise the bar for sensors and qualify for point of care systems. There are limitations to cell-based biosensors such as shelf life, and it will be a challenge for researchers to bring the sensors out of the laboratory to be managed by untrained personnel. However, cell-based biosensors have a unique ability to simulate the physiological *in vivo* response, and will certainly continue to revolutionize sensing of pathogens and toxins in the near future.
